# Combining Neuromuscular Electrical Stimulation with Activity Based Training in Children with Chronic Cervical Spinal Cord Injury: A Retrospective Case Series

**DOI:** 10.3390/children13091234

**Published:** 2026-09-12

**Authors:** Kathryn Noonan-Eaton, Beatrice Ugiliweneza, Andrea L. Behrman, Goutam Singh

**Affiliations:** 1 Pediatric NeuroRecovery Translational Program, Louisville, KY 40202, USA; kathryn.noonan@louisville.edu (K.N.-E.); andrea.behrman@louisville.edu (A.L.B.); 2 Kentucky Spinal Cord Injury Research Center, Louisville, KY 40202, USA; beatrice.ugiliweneza@louisville.edu; 3 Department of Anatomical Sciences and Neurobiology, University of Louisville, Louisville, KY 40202, USA; 4 Department of Neurological Surgery, University of Louisville, Louisville, KY 40202, USA; 5 Kosair for Kids School of Physical Therapy, Spalding University, Louisville, KY 40203, USA

**Keywords:** neuromuscular electrical stimulation, spinal cord injury, upper extremity function, activity-based training

## Abstract

**Highlights:**

**What are the main findings?**

- This case series is among the first to describe a combined upper extremity (UE) activity-based training and wide pulse neuromuscular electrical stimulation (WPS-NMES) program in children with chronic cervical SCI.
- Improvements in UE function were observed in most participants during the multimodal rehabilitation program, particularly in reaching, object-to-mouth activities, and manual dexterity tasks.

**What are the implications of the main findings?**

- No serious or treatment-limiting adverse events were observed among the five participants who completed the intervention incorporating WPS-NMES into activity-based UE rehabilitation.
- These preliminary findings support further investigation of multimodal approach combining WPS-NMES and activity-based therapy as a restorative approach to optimize dosing and evaluate their potential for improving UE and trunk function in children with SCI.

**Abstract:**

Background/Objectives: Neuromuscular electrical stimulation (NMES) is a promising adjunct to activity-based training for individuals with spinal cord injury (SCI). While lower extremity NMES applications are well documented, upper extremity (UE) outcomes remain underexplored in pediatric SCI. The objective of this case series was to evaluate outcomes following a combined NMES and activity-based therapy program targeting the UE in children with chronic SCI. Methods: In this retrospective case series, we analyzed outcomes from five children, ages 4 to 15 years old, with chronic cervical level SCI who completed an individualized activity-based training program with concurrent wide pulse neuromuscular electrical stimulation (WPS-NMES). Participants were children with chronic SCI (≥6 months post-injury). Functional assessments were conducted by pre- and post-intervention. Pre- and post-intervention outcomes were summarized descriptively at the individual case level. Results: Five children with chronic cervical SCI (ages 4–15 years) completed a mean of 54 intervention sessions. All patients demonstrated improved trunk control, with Segmental Assessment of Trunk Control (SATCo) increases of 1–6 points. UE gains on the Peds-NRS were observed in 4/5 patients, primarily in overhead reach and object-to-mouth items. Box and Blocks Test scores improved in 4/5 patients in at least one UE, while one patient remained at 0 bilaterally, and one patient experienced a unilateral decline. One patient demonstrated proximal and trunk improvements without changes in manual dexterity. No unanticipated adverse events were reported. Conclusions: Observed improvements in selected trunk and UE outcomes occurred during participation in a multimodal rehabilitation program incorporating WPS-NMES. These preliminary findings describe functional changes observed during the rehabilitation program and may help inform the design of future controlled studies evaluating multimodal rehabilitation approaches for children with chronic SCI.

## 1. Introduction

Pediatric spinal cord injury (SCI) is a debilitating condition that significantly impacts physical function, independence, and quality of life, despite accounting for less than 4% of annual cases [1]. Specifically, pediatric cervical SCI, representing about 1–2% of these cases [2], leads to major impairments in upper and lower extremity function, trunk control, and autonomic function [3]. These impairments disrupt essential activities of daily living, including play, which is vital for a child’s development [4,5]. For children with tetraplegia, recovery of upper extremity (UE) function is consistently identified as a primary rehabilitation priority due to its direct impact on independence and participation in meaningful activities [5]. Research indicates that trunk control is a critical prerequisite for UE function in individuals with SCI. The trunk acts as a “proximal stabilizer”, providing the necessary foundation for distal mobility, such as reaching, grasping, and manipulating objects [6,7].

While UE and trunk recovery are primary rehabilitation priorities, current clinical strategies often emphasize compensatory techniques, such as bracing or surgical interventions, rather than restorative recovery [8]. In contrast, activity-based restorative therapy (ABRT) is theorized to leverage activity-dependent plasticity through intensive, repetitive, task-specific practice to support neuromuscular recovery above, across, and below the level of injury [9]. UE and trunk focused ABRT may include intensive practice of functional activities, bimanual task training, and task-specific reaching and grasping, with an emphasis on kinematically consistent movements, weight-bearing, task-specific sensory input, and minimization of compensatory strategies [10,11,12,13].

Neuromuscular electrical stimulation (NMES) is frequently used as an adjunct to elicit muscle contractions by stimulating peripheral nerves [14,15]. When paired with voluntary effort, NMES enhances central drive and sensorimotor integration. In adults with subacute and chronic cervical SCI, functional electrical stimulation (FES) and NMES have improved hand function and UE clinical outcomes compared to conventional therapies [16,17,18,19]. However, traditional NMES often utilizes non-physiological motor recruitment by using narrow pulse widths (50–400 μs) and lower frequencies (15–40 Hz), limiting its effectiveness for strengthening and recovery [14,15,20,21]. In contrast, wide pulse neuromuscular electrical stimulation (WPS-NMES) utilizes wider pulse durations (approximately 1000 μs) and higher frequencies (100 Hz), enabling activation of both peripheral and central pathways and promoting a more physiological recruitment pattern [14,15,20]. This approach preferentially targets fatigue-resistant motor units vulnerable after SCI and may enhance the recovery potential of NMES-based interventions [22]. While WPS-NMES has shown promise in adults, its combined use with ABRT in pediatric SCI remains underexplored.

Although emerging literature includes patients who have received NMES targeting the UE [23], to our knowledge, no published studies have specifically examined the combined use of WPS-NMES and ABRT in pediatric SCI. This case series describes the clinical implementation of this combined approach in pediatric patients with chronic cervical SCI. The purpose of this report is to: (1) describe the novel integration of ABRT and WPS-NMES within a pediatric SCI rehabilitation program; (2) examine safety, feasibility, and observed UE and trunk motor changes; and (3) provide preliminary clinical insights to guide future research and clinical application in this underserved population.

## 2. Materials and Methods

This retrospective case series was conducted at the Frazier Rehabilitation Institute (Louisville, KY, USA) over a seven-year period and included five children (three females, two males; ages 4–15 years) with chronic cervical SCI. All patients were non-ambulatory, used strollers or manual/power wheelchairs for mobility, and demonstrated severe UE and trunk impairments (SATCo scores < 15). Patients were identified through the outpatient Pediatric NeuroRecovery Program or recruited via a University of Louisville Institutional Review Board (IRB)-approved research database for potential research volunteers (IRB #06.0647). The legal guardians of current clinical patients signed an IRB- approved informed consent document (IRB 05-016J) allowing their clinical outcomes to be retained for future research and program evaluation. A review of clinical records identified five participants who met all inclusion criteria and had complete pre- and post-intervention data available for analysis. An initial review identified 10 participants who met screening criteria; however, five participants were excluded because their SCI was of perinatal etiology. To reduce heterogeneity and improve clinical comparability, only participants with traumatic SCI were included in the final analysis. Participants were included if they met the following criteria: (1) chronic traumatic SCI (≥6 months post-injury) [24,25] with upper motor neuron (UMN) or mixed UMN/lower motor neuron (LMN) presentations; (2) age 3–18 years; (3) a motor response of at least trace movement to NMES in the majority of the UE muscle groups stimulated; (4) no prior history of WPS-NMES use; (5) completion of a minimum of 34 WPS-NMES ABRT intervention sessions. Patients with a history of surgical interventions (e.g., scoliosis correction, tendon lengthening, or transfers), lower motor neuron (LMN) injuries (e.g., acute flaccid myelitis), comorbidities interfering with participation, ongoing spasticity management (e.g., Botox use within 3 months), and/or ventilator dependence were excluded. Eligibility was confirmed by a clinical physician. Participants are reported using de-identified identifiers (e.g., Publication ID P14) (Table 1).

### 2.1. Outcome Measures

Patients completed approximately 60 sessions (mean = 54 sessions, range = 34–63 sessions) and were evaluated using the following validated outcome measures.

(1) The Pediatric Neuromuscular Recovery Scale (Peds-NRS) measures functional recovery across the upper extremities, trunk and lower extremities in SCI based on pre-injury functional capability [7,23,26]. The Peds-NRS evaluated UE motor recovery using three UE items (object to mouth, overhead reach, and in-hand manipulation) and one trunk item (sit outside BOS), with scores classified into four phases ranging from 1A (no recovery) to 4C (defined recovery) [23,26].

(2) The Segmental Assessment of Trunk Control (SATCo) measures trunk control in sitting across seven levels of support and three control types (static, active, and reactive), progressive from full trunk support (top of shoulder) to no external support [27,28]. Scores range from 0 to 20, with higher scores indicating less support required and greater segmental trunk control.

(3) The Box and Blocks Test (BBT) was used to assess unilateral gross manual dexterity, specifically the ability to repeatedly grasp, transport, and release objects within a 1-min period [29]. The test requires the patient to grasp one-inch wooden cubes individually, transport them across a partition, and release them into an adjacent compartment as quickly as possible, emphasizing repetitive grasping, object manipulation, and coordinated reach across midline. All reported assessment scores were obtained using standard administration and scoring procedures. All baseline outcome measures were performed prior to any intervention, with repeat assessments conducted before the application of WPS-NMES on the day of testing to minimize the influence of acute neuromodulatory effects associated with WPS-NMES [30].

### 2.2. Intervention

All five patients received activity-based occupation training (AB-OT) 5 days a week for 60–90 min with a goal of administering 60 min of WPS-NMES (Xcite; Restorative Therapies, Inc., Baltimore, MD, USA). WPS-NMES was delivered as biphasic rectangular pulses at 100 Hz and 1000 μs pulse duration for 45–60 min during each AB-OT session. In addition to AB-OT, patients were also enrolled in clinic to receive activity-based locomotor training (focus on lower extremities and trunk) daily for 5 days/week and AB-OT was typically administered after AB-LT to capitalize on the proposed increase in central nervous system excitability associated with locomotor and overground training [31].

A multichannel NMES (Xcite; Restorative Therapies, Inc., Baltimore, MD, USA) was utilized to deliver WPS-NMES and was customized per patient using clinical decision making, Peds-NRS lagging items (lowest scoring Peds-NRS UE item), and motor point mapping [14,15]. Stimulation parameters were individualized by the treating therapist based on participant presentation, therapeutic goals, and clinical response. Due to the retrospective nature of this case series and the use of routine clinical documentation, duty cycle and ramp time parameters were not consistently documented and therefore could not be reported retrospectively. Electrode placements varied across patients with placements remaining at the trunk and/or UEs (Table S1). Peripheral WPS-NMES muscle testing guided the initial selection of stimulation channels and parameter settings, with periodic clinical adjustments made throughout the intervention based on participant tolerance and emerging motor responses. This procedure utilized WPS-NMES to evaluate neuromuscular responsiveness to electrical stimulation. Stimulation parameters were iteratively adjusted to identify the minimum intensity required to achieve either participant-tolerated sensory threshold or a visible, isolated muscle contraction. Stimulation intensity was titrated throughout the treatment based on participant tolerance and observed motor response to optimize task-specific muscle activation. Muscle groups were initially assessed individually, followed by multi-channel testing to determine the parameters that produced the most effective and coordinated motor response.

Intervention goals followed a structured, recovery-based progression, guided by the Peds- NRS, with intervention goals targeting the participant’s lowest scoring item(s). This ABRT protocol provided task-specific functional movements to activate the neuromuscular system below the level of injury. The theoretical rationale for providing intensive, repetitive, task-specific sensory and motor input is that such training may enhance central nervous system excitability and support activity-dependent plasticity; however, these neurophysiological mechanisms were not directly assessed in this case series. Task-specific training incorporated structured alternation between stimulated and volitional movements (with and without WPS-NMES) to provide repeated sensory and motor experiences that may support motor learning and transfer of emerging motor responses. Training consisted of meaningful, play-based functional activities that varied in task demands and context, such as building block towers, reaching for and manipulating toys, throwing and catching a ball, and object retrieval tasks, while encouraging appropriate UE and trunk movements with therapist support provided as needed. Manual facilitation and sensory cueing supported motor learning throughout the movement pattern, while stimulation targeted motor points to evoke functional contractions and provide sensory input. Trunk and UE control were progressively challenged in standing or seated tasks to promote postural alignment, load bearing, and UE engagement. Finally, AB-OT emphasized community integration and the generalization of restorative principles across environments through daily parent education and consistent caregiver communication to ensure therapeutic carryover. The total number of intervention sessions varied across patients (average of 54 sessions; Table 2). Despite variability in total session count, session structure, therapeutic intensity, and NMES dosing remained consistent across cases.

Standardized Elements: Stimulation frequency (100 Hz), pulse duration (1000 μs), general session structure, target session duration (60–90 min), and treatment frequency (5 days/week) were held constant across all participants. Individualized Elements: Stimulation intensity, electrode placement sites, targeted muscle groups, functional task progression, and total session count (34–63 sessions) were customized based on individual baseline presentation and clinical tolerance.

### 2.3. Individual Case Presentations

Case 1 P14 was a 7-year-old male enrolled approximately 4 years after sustaining a traumatic cervical SCI complicated by multilevel cord edema and prior spinal fusion (Table 1). He completed 59 sessions with electrical stimulation intensity ranging from 1–20 mA, the highest range across participants, targeting forward reaching and grasp (Table 2).

Case 2 P235 was a 4-year-old male enrolled approximately 6 months after sustaining a traumatic cervical SCI characterized by C2–C3 subluxation, C4–C5 fracture, unilateral left C4–C5 perched facet, and associated spinal cord hematoma (Table 1). He completed 60 sessions with electrical stimulation intensity ranging from 3–19 mA, targeting forward and overhead reaching and grasp (Table 2).

Case 3 P279 was a 15-year-old female enrolled approximately 2 years after sustaining a traumatic cervical SCI involving a C3–C4 fracture with associated hematoma (Table 1). She completed 63 sessions with electrical stimulation intensity ranging from 1–8 mA, the lowest range across participants due to increased sensory responsiveness, targeting forward reach and grasp (Table 2).

Case 4 P278 was a 6-year-old female enrolled approximately 3.5 years after sustaining a traumatic cervical SCI characterized by a C6–C7 fracture with ligamentous injury (Table 1). She completed 56 sessions with electrical stimulation intensity ranging from 1–13 mA, targeting forward reaching and grasp (Table 2).

Case 5 P8 was a 7-year-old female enrolled approximately 4.5 years after sustaining a traumatic cervical SCI, including a C2 fracture with C1–C2 subluxation requiring spinal fusion and additional involvement at C6–C7 (Table 1). She completed 34 sessions, fewer than other participants due to non-intervention factors, with electrical stimulation intensity ranging from 1–13 mA and targeting forward and overhead reaching and grasp (Table 2).

## 3. Results

Five patients with chronic cervical SCI (ages 4 years to 15 years) completed pre- and post-intervention assessments following participation in combined ABRT and WPS-NMES program. All patients were non-ambulatory and demonstrated substantial UE and trunk impairments. There was a higher ratio of females to males (60% female, 40% male), with all participants having sustained a traumatic SCI. All participants were in the chronic phase of injury (≥6 months post-SCI), with 80% (4/5) enrolled >1 year post-SCI and 20% enrolled during the early chronic phase (6–12 months post-SCI). All patients received a combination of AB-OT and WPS-NMES at a frequency of 100 Hz and a pulse width of 1000 μs (Table 1). Outcome measures included the Peds-NRS, SATCo, and BBT. Across all intervention sessions completed by the five participants, no unanticipated adverse events were observed. Minor transient skin erythema at electrode sites was occasionally noted following stimulation, which resolved within approximately one hour without intervention. No serious or treatment-limiting adverse events were observed during the recorded intervention sessions, and no participant discontinued treatment or required intervention modification due to stimulation-related discomfort.

P14 demonstrated improvement in overhead reaching (Left: 2C to 3A) (e.g., Peds NRS cards/tables) and SATCo score (7 to 12), with Box & Blocks gains bilaterally (L: 19 to 21; R: 23 to 28), suggesting observed improvements in proximal and distal UE function following the multimodal intervention (Table 3).

P235 showed improvements in bilateral overhead reaching (L: 1B to 3A; R: 1B to 2C), bilateral object-to-mouth (1C to 2C), and SATCo score (9 to 11). Notable improvements were observed in Box & Blocks bilaterally (L: 6 to 20; R: 8 to 11), indicating observed increases in manual dexterity alongside improvements in proximal control and sitting balance (Table 3).

P279 demonstrated improvements in SATCo score (5 to 7) and left-side object-to-mouth (1C to 2B) (e.g., Peds-NRS cards/tables), while in-hand manipulation and overhead reach remained the same. Box & Blocks scores remained at 0 bilaterally, suggesting that while trunk control and some proximal functions improved, distal functional gains were limited (Table 3).

P278 showed a substantial increase in SATCo score (5 to 11) and Box & Blocks on the right (12 to 38), along with an increase in the left Box & Blocks score (9 to 31). While object-to-mouth and in-hand manipulation remained the same, the observed improvements in bilateral dexterity and trunk control were associated with changes in UE function (Table 3).

P8 demonstrated improved SATCo score (7 to 9) and a significant increase in Box & Blocks on the right (34 to 45), though they were the only patient to show a slight decline on the left (4 to 3). Most Peds-NRS measures, including overhead reach and object-to-mouth, remained the same, suggesting the intervention’s impact was most pronounced in trunk stability and right-hand manual dexterity (Table 3).

## 4. Discussion

This case series describes observed changes in trunk and UE function during participation in a multimodal rehabilitation program incorporating ABRT and WPS-NMES in children with cervical SCI. Across the five participants, improvements in segmental trunk control were observed, as evidenced by changes in SATCo scores. UE changes were also observed, with four of five participants demonstrating an increase in the number of blocks transported on the BBT, along with observed changes in proximal reaching and manual dexterity. Because participants received concurrent AB-LT alongside AB-OT + WPS-NMES, the overall intervention represents a multimodal rehabilitation program. However, the specificity of training inputs must be considered when interpreting task-domain outcomes. AB-LT primarily engages lower extremity and trunk neural pathways to promote posture, stepping, and axial stability: thus, observed improvements in trunk control (e.g., SATCo scores) likely reflect the collective contribution of AB-LT and AB-OT + WPS-NMES. In contrast, AB-LT does not incorporate UE task training or distal UE activation. Consequently, observed changes in UE reaching, object manipulation, and manual dexterity (e.g., Peds-NRS UE items and BBT) are most likely related to the UE targeted AB-OT + WPS-NMES intervention, as these domains were not directly addressed during concurrent AB-LT.

### 4.1. Trunk Control as a Foundational Domain

Improvements in trunk control were observed in all five patients, as measured by the SATCo and Peds-NRS Sit-outside base of support item. Progression across these measures reflected changes in the level of trunk support required and the ability to demonstrate active trunk control during assessed tasks. For example, P235 progressed from maintaining head alignment during externally induced tilt to actively returning to upright from 20 degrees of trunk flexion. P278 advanced from reactive upper thoracic to reactive lower thoracic control, reflecting changes in distal trunk segment control. These gains are particularly noteworthy given the chronicity of injury and the importance of proximal postural stability for tasks involving UE use. Although functional activities such as play, self-feeding, and tabletop tasks were observed clinically during rehabilitation, these activities were not formally assessed as outcome measures; therefore, relationships between changes in trunk control and functional performance cannot be determined from this case series. Improved trunk control may be relevant to participation in activities requiring postural stability and proximal support for upper extremity use, but this relationship warrants prospective investigation using standardized functional measures.

### 4.2. Heterogeneity in Upper Extremity Response

UE outcomes, as measured by the Peds-NRS Reach Overhead, Object-to-Mouth, and In-Hand Manipulation items, demonstrated heterogeneous changes across participants. Several participants progressed from initiating elbow flexion with compensatory shoulder abduction and limited forearm pronation to completing elbow and shoulder flexion with more appropriate movement patterns, as reflected by advancement across Peds-NRS classifications.

Object-to-Mouth performance was largely stable across participants; however, changes in movement patterns reflected by progression in Peds-NRS classifications were observed in some participants. These observations highlight the potential value of examining movement quality in addition to ordinal outcome scores when evaluating UE function in children with SCI.

Manual dexterity, as assessed by the Box and Blocks Test, demonstrated observed score increases in the majority of participants. Participants with emerging distal motor control demonstrated the largest absolute gains, while those with limited distal activation showed less change, including one participant who remained at zero bilaterally and one who demonstrated a unilateral decline. These heterogeneous responses underscore the variability in functional changes observed across participants.

Standardized outcome measures may not fully capture qualitative changes in movement performance. For example, progression between adjacent Peds-NRS classifications may reflect changes in the quality or execution of a movement that are not captured by a large numerical score change. However, the clinical significance of these ordinal changes cannot be determined from this case series in the absence of established responsiveness thresholds or clinically important difference values. Future studies should incorporate outcome measures capable of capturing both quantitative changes in performance and qualitative aspects of movement quality and functional participation. While several participants demonstrated potentially clinically relevant observed changes, the clinical significance of these findings cannot be determined from this case series given the absence of established responsiveness thresholds or clinically important difference values for these measures. Individual variability exists and may be related to clinical characteristics; however, this is a limitation of this study, and future studies should compare and perform analysis to identify a new relationship between these variables.

### 4.3. Parent Engagement and Environmental Context

Although not systematically quantified in this case series, informal, anecdotal observations suggested that parent engagement, session attendance, and carryover of strategies into home and community settings varied across families and may have been relevant to differences in outcomes. P279, who demonstrated the least progress, experienced documented logistical barriers to consistent therapy attendance and difficulty implementing home programs. Conversely, P278, who showed substantial gains, had consistent parental involvement and reported integration of stimulation-facilitated activities into daily routines. These observations are anecdotal and should be considered hypothesis-generating rather than explanatory evidence. These observations align with the broader early intervention literature, which emphasizes that child outcomes are shaped not only by the intervention itself but also by the quality of parent–child interaction, family adherence, and the degree to which therapeutic strategies are embedded into naturalistic environments [32]. While the present case series did not include a formal measure of parent engagement, these patterns underscore the potential importance of family-centered coaching models in future pediatric NMES-ABRT protocols.

### 4.4. Intervention Characteristics and Stimulation Parameters

The intervention combined high-repetition, task-specific ABRT with concurrent WPS-NMES. ABRT utilized task-specific functional movements to activate the neuromuscular system below the level of injury. By providing task-specific sensory cues during intensive, repetitive training, this approach aimed to optimize central nervous system excitability and promote activity-dependent plasticity for motor recovery. Unlike conventional NMES (narrow pulse widths, 50–400 μs), WPS-NMES employs wider pulse durations (1000 μs) and higher frequencies (100 Hz), which are hypothesized to engage both peripheral and central pathways via sensory afferent activation, promoting more physiological, fatigue-resistant recruitment [15,20]. Stimulation parameters were held constant (100 Hz, 1000 μs) while intensity was titrated individually to evoke functional, non-painful contractions. Electrode placement and task progression were adapted per session based on real-time motor response. By pairing stimulation with voluntary, task-specific effort, the intervention aimed to reinforce activity-dependent plasticity and carryover to volitional movement [33]. Patients with even partial distal motor control at baseline (e.g., P235, P278) demonstrated the largest gains on the Box and Blocks Test, a pattern that in light of previous literature, raises the hypothesis that residual corticospinal connectivity may contribute to the ability to leverage WPS-NMES mediated neuromodulation. However, corticospinal connectivity was not directly assessed in this case series. Conversely, patients with minimal distal activation (P279) showed primarily proximal and trunk improvements. This pattern aligns with evidence that WPS-NMES facilitated contractions rely, in part, on intact central drive [34]. Session frequency (5 days/week) and total volume (average 54 sessions) exceeded typical outpatient dosing, a likely contributor to outcomes but also a limitation to generalizability. Future studies should examine dose-response relationships and standardize electrode montages while preserving clinical flexibility.

### 4.5. Feasibility and Safety of WPS-NMES

While concurrent AB-LT provided continuous activation of trunk and postural networks, UE functional recovery requires task-specific motor activation and sensory feedback unique to the upper limb. The selective gains observed in overhead reaching, object-to-mouth execution, and manual dexterity directly reflect the targeted inputs delivered during AB-OT + WPS-NMES. Rather than acting as a confound for UE gains, AB-LT likely served as an axial foundation, improving proximal trunk stability, which is an established physiological prerequisite for optimizing UE reaching and distal manual control. Together, these findings support the feasibility and potential functional relevance of combining ABRT with WPS-NMES in children with chronic spinal cord injury. Improvements in trunk control appear to provide a critical foundation for UE function, upright seated ability enables a dynamic base for reaching, grasping, and object manipulation, a relationship documented in adults [35,36], and in children with neuromotor impairment [23,37]. These results are consistent with adult SCI literature demonstrating that WPS-NMES can engage both peripheral and central motor pathways, enhancing voluntary motor output and functional task performance when paired with task-specific training [15,18,19,20,38]. The present observations provide preliminary pediatric case series data that are broadly consistent with findings reported in adult SCI populations. Although participants with residual distal motor control demonstrated greater gains on the Box and Blocks Test, this pattern should be interpreted cautiously, as corticospinal connectivity was not directly measured. Differences in baseline functional status, injury severity, age, time since injury, intervention exposure, adherence, and individual neurological heterogeneity may also have contributed to the observed variability in outcomes. Importantly, no serious or treatment-limiting adverse events were observed during the recorded intervention sessions, including no episodes of autonomic dysreflexia, skin breakdown, or stimulation intolerance, findings that are particularly notable given the vulnerability of this population to stimulation-related complications. These results provide initial evidence supporting the safety and clinical feasibility of WPS-NMES combined with ABRT in children with severe and chronic cervical SCI.

### 4.6. Limitations

This case series has several limitations. First, the small sample size and observational design limit generalizability, and the absence of a control group precludes causal attribution. Second, all participants received concurrent daily activity-based locomotor training (AB-LT) in addition to AB-OT and WPS-NMES. While AB-LT specifically targets lower extremity and trunk circuitry, making it difficult to isolate the contribution of AB-LT to observed trunk improvements, it does not include UE specific training. Therefore, while trunk changes represent a combined multimodal effect, UE functional gains remain attributable to the UE-focused AB-OT and WPS-NMES intervention. Furthermore, previous and ongoing rehabilitation services may have contributed to observed outcomes and could not be controlled for in this retrospective case series. Third, the requirement that participants complete a minimum of 34 intervention sessions may have introduced a selection bias, as participants who were able to meet this threshold may have had greater treatment tolerance, family support, access to care, or adherence than those unable to complete the required sessions. In addition, total treatment exposure, stimulation intensity, electrode placement, and task progression varied across participants based on clinical presentation and response to therapy, which may have contributed to variability in outcomes. Duty cycle and ramp time settings were also individualized during routine clinical care and were not systematically documented, preventing retrospective reporting of these parameters. This criterion may also have resulted in a sample that was not representative of the broader pediatric SCI population. Fourth, heterogeneity in age, injury level, and chronicity introduces variability that may influence responsiveness to therapy. Although all participants met the standard criterion for chronic SCI (≥ 6 months post-onset), P235 initiated intervention at approximately 6 months post-injury. While classified as chronic, spontaneous neurological recovery cannot be ruled out as a contributing factor for this participant, which further limits causal interpretation. Given the sample size and heterogeneity of the cohort, we were unable to determine whether specific clinical characteristics were associated with treatment responsiveness or identify subgroups most likely to benefit from the intervention. Fifth, certain outcome measures, particularly fine motor assessments, were not feasible for all patients due to age or functional limitations, potentially underestimating intervention effects. Sixth, the lack of a formal measure of parent engagement or treatment fidelity limits our ability to examine the contribution of environmental factors to outcomes. Finally, post-intervention follow-up assessments were not collected consistently across participants, limiting our ability to evaluate the maintenance of treatment effects over time. Clinical outcome measures may not fully capture underlying neurophysiological changes associated with recovery.

### 4.7. Future Directions

While patients in this case series demonstrated meaningful improvements after an average of 54 sessions, several children were still progressing at discharge. This suggests that extending the duration of therapy may yield further gains, and future studies should systematically investigate the dose–response relationship for UE recovery in pediatric SCI. Future studies should prioritize prospective, controlled designs with predefined case selection criteria, standardized intervention dosing, clear treatment fidelity monitoring, repeated outcome assessments, and long-term follow-up. Additionally, systematically evaluating dose-response relationships and combining WPS-NMES with other non-invasive modalities, such as transcutaneous spinal cord stimulation (scTS), may further inform treatment optimization and functional recovery in pediatric SCI [39,40]. Combining NMES with scTS has shown promise in enhancing motor cortex excitability in adults [41] and may represent a powerful, non-invasive strategy to augment UE recovery in children. Future research should also prioritize the development of sensitive, pediatric-specific outcome measures capable of detecting qualitative improvements in movement quality and functional participation.

## 5. Conclusions

In this retrospective case series, improvements in selected trunk and UE outcomes were observed during participation in a multimodal rehabilitation program incorporating AB-LT, AB-OT, and WPS-NMES. Because of the small heterogeneous sample, concurrent therapies, variable intervention exposure, and absence of a comparator, the contribution of WPS-NMES to these changes cannot be isolated. These observations support further prospective investigation of feasibility, dosing, and functional outcomes.

## Figures and Tables

**Table 1:** Patient Demographics and Clinical Characteristics.

Participant	Gender	SCI Type	Age at Onset	Age at Enrollment	AIS	Previous Therapies
P14	M	Traumatic	3 y 6 m	7 y 5 m	C7 A	Inpatient & outpatient OT and PT
P235	M	Traumatic	3 y 6 m	4 y	C1	Inpatient & outpatient OT and PT
P279	F	Traumatic	13 y 7 m	15 y 6 m	C3 B	Inpatient & outpatient OT and PT
P278	F	Traumatic	2 y 9 m	6 y 5 m	C1 A	Inpatient & outpatient OT and PT
P8	F	Traumatic	2 y 4 m	7 y	C3 A	Inpatient & outpatient OT and PT

SCI = Spinal Cord Injury; OT = Occupational Therapy; PT = Physical Therapy; y = years; m = months; C = cervical; T = Thoracic; AIS= American Spinal Injury impairment scale.

**Table 2:** Activity Based Restorative Therapy Intervention Characteristics.

Participant	Frequency	Pulse Duration	Intensity Range	Targeted Motor Task/Goal	Session Duration	NMES Duration	Sessions/ Week	Total Sessions
P14	100 Hz	1000 μs	1–20 mA	Forward reach and grasp	60–90 min	45–60 min	5	59 sessions
P235	100 Hz	1000 μs	3–19 mA	Overhead & forward reach and grasp	60–90 min	45–60 min	5	60 sessions
P279	100 Hz	1000 μs	1–8 mA	Forward reach and grasp	60–90 min	45–60 min	5	63 sessions
P278	100 Hz	1000 μs	1–13 mA	Forward reach and grasp	60–90 min	45–60 min	5	56 sessions
P8	100 Hz	1000 μs	1–13 mA	Overhead & forward reach and grasp	60–90 min	45–60 min	5	34 sessions

NMES = Neuromuscular Electrical Stimulation; Hz = frequency; μs = microseconds; mA = milliampere; All participants received 100 Hz frequency and 1000 μs pulse duration.

**Table 3:** Clinical Outcomes: Pre- and Post- Activity Based Restorative Therapy Intervention.

Clinical Outcome Measure	P14		P235		P279		P278		P8	
	Pre	Post	Pre	Post	Pre	Post	Pre	Post	Pre	Post
Object to Mouth R	2B	2B	1C	2C	2C	2C	2C	2C	2B	2B
Object to Mouth L	2C	2C	1C	2C	1C	2B	2C	2C	4C	4C
In-Hand Manipulation L	1B	1B	1B	1B	1B	1B	1B	1B	1B	1B
In-Hand Manipulation R	1B	1B	1B	1B	1A	1A	1B	1B	4C	4C
Overhead Reach L	2C	3A	1B	3A	1B	1B	3A	3B	2A	2A
Overhead Reach R	3A	3A	1B	2C	1B	1B	3A	3C	4C	4C
Sit Outside Base of Support	2B	2C	1C	2A	1A	1A	1C	1C	1C	1C
SATCo (out of 20)	7	12	9	11	5	7	5	11	7	9
Box & Blocks L (number of blocks)	19	21	6	20	0	0	9	31	4	3
Box & Blocks R (number of blocks)	23	28	8	11	0	0	12	38	34	45

R–Right, L = Left, NT = Not Tested. SATCo = Segmental Assessment of Trunk Control; Box & Blocks = Box and Block Test (number of blocks transferred).

## Data Availability

The data presented in this study are not publicly available due to privacy and ethical restrictions, as the dataset contains protected health information from pediatric patients. De-identified outcome data supporting the findings of this case report are reported within the article. Additional information may be available from the corresponding author upon reasonable request and subject to institutional and ethical approval requirements.

## Supplementary Materials

The following supporting information can be downloaded at: https://www.mdpi.com/article/10.3390/children13091234/s1, Table S1: Participant specific stimulation parameters and training characteristics.

## Abbreviations

The following abbreviations are used in this manuscript:

SCI	Spinal Cord Injury
WPS	Wide pulse width
NMES	Neuromuscular electrical stimulation
UE	Upper extremity
ABRT	Activity-based restorative therapy
IRB	Institutional review board
Peds-NRS	Pediatric Neuromuscular Recovery Scale
SATCo	Segmental Assessment of Trunk Control
BBT	Box and Blocks Test
AB-OT	Activity-based occupation training
scTS	Transcutaneous spinal cord stimulation
