# Supplementary material for: Combining Neuromuscular Electrical Stimulation with Activity Based Training in Children with Chronic Cervical Spinal Cord Injury: A Retrospective Case Series
Source: Children (Basel). 2026 Sep 12;13(9):1234. doi: 10.3390/children13091234 (PMC13605759; doi:10.3390/children13091234)
Supplement: Supplementary file 1 [file children-13-01234-s001.zip › children-4480968-supplementary.pdf]

Supplementary Table S1: Participants specific stimulation parameters and training characteristics.

Participant	Intensity Range	Targeted Motor Task/Goal	Session Duration	Electrode Location (Left)	Electrode Location (Right)	NMES Duration	Sessions/ Week	Total Sessions
**P14**	1–20 mA	Forward reach and grasp	60–90 min	Muscle 1: Finger Flexors<br>Muscle 2: Hypothenar/Thenar<br>Muscle 3: Oblique<br>Muscle 4: Erector Spinae<br>Muscle 5: Upper Rectus Abdominis	Muscle 1: Finger Flexors<br>Muscle 2: Hypothenar/Thenar<br>Muscle 3: Oblique<br>Muscle 4: Erector Spinae<br>Muscle 5: Upper Rectus Abdominis	45–60 min	5	59
**P235**	3–19 mA	Overhead & forward reach and grasp	60–90 min	Muscle 1: Hypothenar/Thenar<br>Muscle 2: Wrist Extensors<br>Muscle 3: Wrist Extensors<br>Muscle 4: Wrist Flexors<br>Muscle 5: EPL	Muscle 1: Hypothenar/Thenar<br>Muscle 2: Wrist Extensors<br>Muscle 3: Wrist Extensors<br>Muscle 4: Wrist Flexors<br>Muscle 5: EPL	45–60 min	5	60
**P279**	1–8 mA	Forward reach and grasp	60–90 min	Muscle 1: Wrist Flexors<br>Muscle 2: Hypothenar/ thenar<br>Muscle 3: Extensor Pollicis Longus	Muscle 1: Wrist Flexors<br>Muscle 2: Hypothenar/ thenar<br>Muscle 3: Extensor Pollicis Longus<br>Muscle 4: Triceps<br>Muscle 5: Biceps<br>Muscle 6: Anterior Deltoid	45–60 min	5	63
**P278**	1–13 mA	Forward reach and grasp	60–90 min	Muscle 1: Thenar right<br>Muscle 2: Finger Extensors<br>Muscle 3: Wrist Flexors<br>Muscle 4: Scapular Stabilizers<br>Muscle 5: Erector Spinae<br>Muscle 6: Dorsal Interossei	Muscle 1: Thenar right<br>Muscle 2: Finger Extensors<br>Muscle 3: Wrist Flexors<br>Muscle 4: Scapular Stabilizers<br>Muscle 5: Erector Spinae<br>Muscle 6: Dorsal Interossei	45–60 min	5	56
**P8**	1–13 mA	Overhead & forward reach and grasp	60–90 min	Muscle 1: Thenar<br>Muscle 2: Finger Extensors<br>Muscle 3: Wrist Flexors<br>Muscle 4: Serratus Anterior<br>Muscle 5: Erector Spinae<br>Muscle 6: Infraspinatus	Muscle 1: Thenar right<br>Muscle 2: Finger Extensors<br>Muscle 3: Erector Spinae	45–60 min	5	34

NMES = Neuromuscular Electrical Stimulation; mA = milliampere. For all participants, stimulation was delivered at a constant frequency of 100 Hz and pulse duration of 1000 μs.
